# A Pyroptosis-Related Gene Panel for Predicting the Prognosis and Immune Microenvironment of Cervical Cancer

**DOI:** 10.3389/fonc.2022.873725

**Published:** 2022-04-29

**Authors:** Haoran Hu, Meiqin Yang, Wei Dong, Bo Yin, Jianyi Ding, Baoyou Huang, Qingliang Zheng, Fang Li, Lingfei Han

**Affiliations:** ^1^ Department of Gynecology, Shanghai First Maternity and Infant Hospital, School of Medicine, Tongji University, Shanghai, China; ^2^ Department of Gynecology, The First Affiliated Hospital of Wenzhou Medical University, Wenzhou, China; ^3^ Prenatal Diagnosis Center, The Eighth Affiliated Hospital, Sun Yat-sen University, Shenzhen, China; ^4^ Department of Gynecology, Shanghai East Hospital, School of Medicine, Tongji University, Shanghai, China

**Keywords:** pyroptosis-related genes, panel, tumor immune microenvironment, cervical cancer, prognosis

## Abstract

Cervical cancer (CC) is one of the most common malignant tumors of the female reproductive system. And the immune system disorder in patients results in an increasing incidence rate and mortality rate. Pyroptosis is an immune system-related programmed cell death pathway that produces systemic inflammation by releasing pro-inflammatory intracellular components. However, the diagnostic significance of pyroptosis-related genes (PRGs) in CC is still unclear. Therefore, we identified 52 PRGs from the TCGA database and screened three Differentially Expressed Pyroptosis-Related Genes (DEPRGs) in the prognosis of cervical cancer: CHMP4C, GZMB, TNF. The least absolute shrinkage and selection operator (LASSO) regression analysis and multivariate COX regression analysis were then used to construct a gene panel based on the three prognostic DEPRGs. The patients were divided into high-and low-risk groups based on the median risk score of the panel. According to the Kaplan-Meier curve, there was a substantial difference in survival rates between the two groups, with the high-risk group’s survival rate being significantly lower than the low-risk group’s. The PCA and t-SNE analyses revealed that the panel was able to differentiate patients into high-and low-risk groups. The area under the ROC curve (AUC) shows that the prognostic panel has high sensitivity and specificity. The risk score could then be employed as an independent prognostic factor using univariate and multivariate COX regression analyses paired with clinical data. The analyses of GO and KEGG functional enrichment of differentially expressed genes (DEGs) in the high-and low-risk groups revealed that these genes were primarily engaged in immune response and inflammatory cell chemotaxis. To illustrate immune cell infiltration in CC patients further, we used ssGSEA to compare immune-related cells and immune pathway activation between the high-and low-risk groups. The link between three prognostic DEPRGs and immune-related cells was still being discussed after evaluating immune cell infiltration in the TCGA cohort with “CIBERSORT.” In addition, the GEPIA database and qRT-PCR analysis were used to verify the expression levels of prognostic DEPRGs. In conclusion, PRGs are critical in tumor immunity and can be utilized to predict the prognosis of CC.

## Introduction

Cervical cancer (CC) is the fourth most common and lethal female malignant tumor in the globe. And CC has been the second leading cause of cancer death in women aged 20 to 39. (1). It is well acknowledged that persistent infection of high-risk human papillomavirus (HPV) is necessary for the occurrence and development of CC. Epidemiological studies have shown that nearly 100% of CC cases are positive for HPV, of which the HPV16 and HPV18 subtypes are the most closely linked. However, the coverage rate of HPV vaccination uptake and Papanicolaou/HPV DNA co-testing is still very low (1, 2). For CC, the slow-growing squamous lesions were removed by long-term extensive screening, while the incidence of adenocarcinoma showed an upward or stable trend. Meanwhile, the early downward trend of the incidence of squamous cell carcinoma was weakened, and the incidence of cancer increased in the distant stage (3). In many countries, neoadjuvant chemotherapy (NACT) and radical surgery (RS) are employed as effective options to locally advanced CC. However, approximately 20% to 30% of patients treated with NACT+RS experience pelvic and/or extra-pelvic recurrence within 5 years, and the long-term overall survival (OS) rate remains unsatisfactory (4, 5). As a result, it is critical to investigate effective biomarkers and create innovative prognostic panels for CC.

Host cell death, which includes apoptosis, programmed necrosis, and pyroptosis, is essential for hosts to resist infection and eliminate intracellular pathogens (6, 7). Pyroptosis was first proposed by Cookson and Brenna in 2001 and named caspase-1-dependent cell death to describe atypical death of macrophages infected with Salmonella or Shigella (8). Later research discovered that bacterial infection is not the only cause of this type of cell death, and pyroptosis shares morphological hallmarks of both necrosis and apoptosis (9). Pyroptosis is now widely considered to be a lytic form of regulatory cell death (RCD) caused by inflammasome activation and strongly reliant on the creation of plasma membrane holes mediated by the gasdermin (GSDM) protein family (10). There is widespread agreement that there are two mechanisms for pyroptosis: one non-classical pathway driven by Caspase-4/5/11 activation, and one classical pathway mediated by Caspase-1 activation (11). Pyroptosis is distinguished by the absence of DNA fragmentation in favor of a specific type of chromatin condensation mediated by gasdermin-D (GSDMD). It is cleaved by inflammatory caspase-1 or caspase-11, resulting in extracellular release of IL-1 and IL-18, cell swelling and lysis, and the formation of apoptotic body-like cell protrusions (12). To sum up, cell pyroptosis is a programmed cell death pathway that is essential for immunity, which causes systemic inflammation by releasing pro-inflammatory intracellular contents. Therefore, pyroptosis plays a key role in a variety of diseases, including, but not limited to, sepsis, Alzheimer’s disease (AD), human immunodeficiency virus (HIV), and gout (13). In recent years, there has been an increasingly in-depth study of the correlations between pyroptosis and tumors. It has been reported that it is related to liver cancer (14), triple negative breast cancer (15), ovarian cancer (16), endometrial cancer (17), gastrointestinal cancer (18) and so on. According to some research, HPV E7 reduces cell pyroptosis by enhancing TRIM21-mediated degradation and ubiquitination of the IFI16 inflammasome (19), while MiRNA-214 promotes pyroptosis and decreases CC cell proliferation *via* modulating NLRP3 expression (20). Tanshinone II A induces cervix cancer cell death *via* the miR-145/GSDMD signal pathway and carboplatin *via* the caspase-3/GSDME signal pathway (21, 22). These studies show that pyroptosis is closely related to the occurrence, development and treatment of CC. However, the research is limited to this, and there are few studies on the specific function and mechanism of pyroptosis in CC.

Therefore, we conducted a systematic study by using TCGA database to analyze the expression of PRGs in normal cervical and CC tissues, and then constructed a panel based on these genes that is able to independently predict the prognosis of patients with CC. Finally, we studied the immune microenvironment of patients with CC and the correlation with pyroptosis.

## Materials and Methods

**Figure 1:**
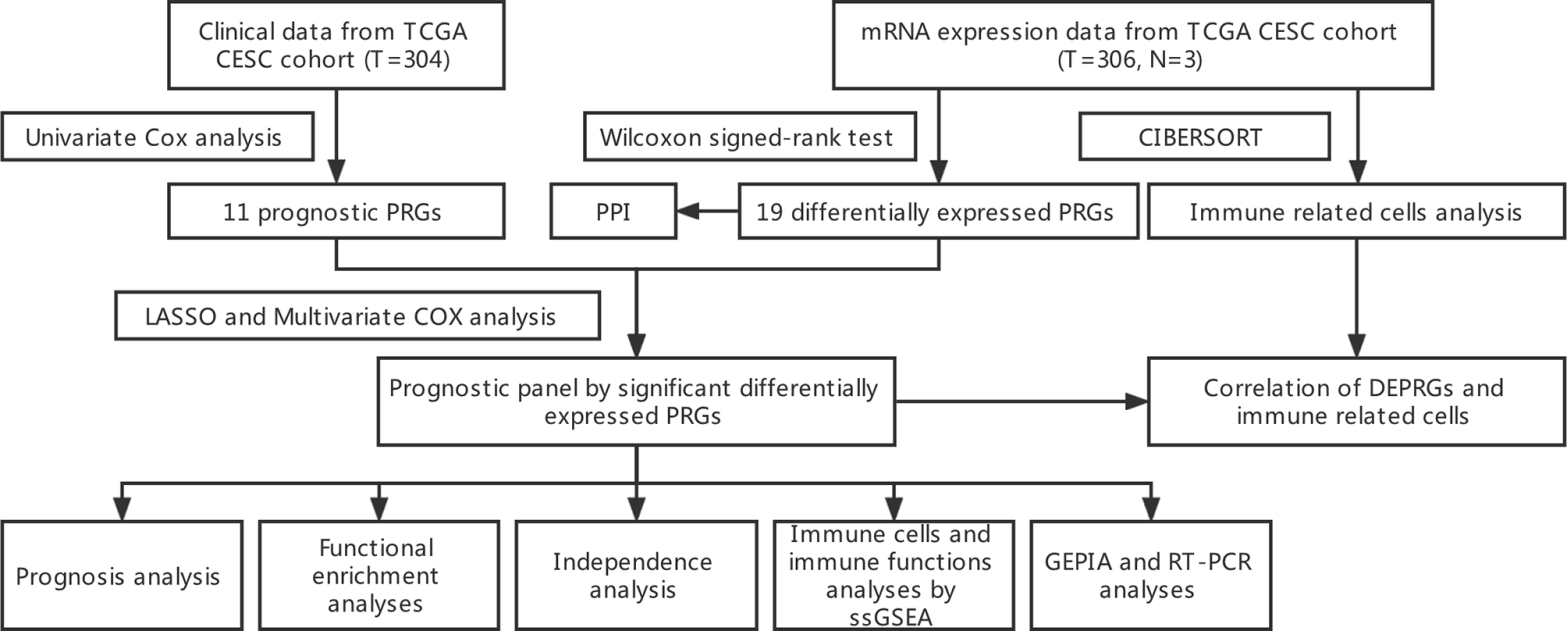
Workflow diagram. The workflow graph of whole process of data analysis.

The flow chart in **Figure 1** depicts the data analysis process.

### mRNA Expression Data and Clinical Data From TCGA CESC Cohort

The mRNA expression data and clinical data of CESC (Cervical squamous cell carcinoma and endocervical adenocarcinoma) patients were retrieved from the TCGA website on November 07, 2021 (https://portal.gdc.cancer.gov/). The mRNA expression data includes 306 CESC samples and 3 normal cervical samples, and the clinical characteristics of patients were shown in **Table 1**.

**Table 1:** Clinical data.

**NO. of patients**		**304**
Age (years)	≤50	186 (61.2%)
	>50	118 (38.8%)
Histological grade	I+ II	153 (50.3%)
	III+IV	119 (39.2%)
	Unknown	32 (10.5%)
Clinical stage	I+ II	230 (75.6%)
	III+IV	68 (22.4%)
	Unknown	6 (2.0%)
Survival status	Alive	233 (76.6%)
	Dead	71 (23.4%)

Clinical characteristics of patients with cervical cancer in TCGA.

### Identification of Prognostic Differentially Expressed PRGs

PRGs were obtained through literature mining (23, 24). Using a false discovery rate (FDR)<0.05, the “limma” package was used to find Differentially Expressed Genes (DEGs). Differentially Expressed Pyroptosis-Related Genes (DEPRGs) are found at the junction of DEGs and PRGs. A PPI network for the DEGs was constructed to show the interactions of the DEPRGs with Search Tool for the Retrieval of Interacting Genes (STRING) (https://string-db.org/). And a network analysis of internal correlations among DEPRGs realized by “igraph” and “reshape2” R packages. To screen for prognostic PRGs, we used a univariate Cox analysis of OS. The intersection of DEPRGs and prognostic PRGs are the prognostic DEPRGs. All the related genes are shown in **Table 2**.

**Table 2:** Genes Selection 52 pyroptosis-related genes, 19 differentially expressed PRGs, 11 prognostic PRGs, and 3 DEPRGs.

52 pyroptosis-related genes	BAK1 BAX CASP1 CASP3 CASP4 CASP5 CHMP2A CHMP2B CHMP3 CHMP4A CHMP4B CHMP4C CHMP6 CHMP7 CYCS ELANE GSDMD GSDME GZMB MGB1 IL18 IL1A IL1B IRF1 IRF2 TP53 TP63 AIM2 CASP6 CASP8 CASP9 GPX4 GSDMA GSDMB GSDMC IL6 NLRC4 NLRP1 NLRP2 NLRP3 NLRP6 NLRP7 NOD1 NOD2 PJVK PLCG1 PRKACA PYCARD SCAF11 TIRAP TNF GZMA
19 differentially expressed PRGs	BAK1 BAX CASP3 CASP5 CHMP4C CHMP6 CYCS ELANE GZMB IL18 AIM2 CASP6 CASP8 GSDMB GSDMC NLRP7 NOD2 PYCARD TNF
11 prognostic PRGs	CHMP4C CHMP7 GZMB IL1A IL1B TP53 GPX4 NOD1 PRKACA TNF GZMA
3 significant differentially expressed PRGs	CHMP4C GZMB TNF

### Construction and Validity Analysis of Prognostic Panel Constructed by DEPRGs

The LASSO regression analysis and multivariate COX regression model implemented through the “glmnet” R package were used to construct the panel of DEPRGs (25), and the three DEPRGs and their correlation coefficients were shown in **Table 3**. The penalty parameter (λ) of LASSO regression was determined by the minimum criteria. After the construction of the panel, the risk value of each patient could be obtained by the following formula: $\text{Risk Score}=\Sigma_{i}^{3}Xi\times Yi$ (X: coefficients, Y: gene expression level). Using the “rms” R package (26), a nomogram diagram of the prognostic DEPRGs was constructed for prediction of the 1-, 2-, and 3-year survival rate. According to the median risk score of the panel, the patients were divided into high-and low-risk groups, and a “bioRiskPlot” function was defined to draw the risk curve and survival state plot. The dimensionality reduction of the 3-gene signature was carried out through the PCA analysis by “Rtsne” R package. The “survival” and “survminer” R packages were used to analyze the Kaplan-Meier survival of patients between high-and low-risk groups. At the same time, the “timeROC”, “survival” and “survminer” R packages were used to draw the1-, 2-, and 3-year ROC curve for evaluating the accuracy of the panel prediction (27).

**Table 3:** Prognostic DEPRGs differences.

Gene	Coef	LogFC	P-value	FDR
CHMP4C	0.420	3.404	0.003	0.036
GZMB	-0.224	3.756	0.016	0.047
TNF	0.457	3.456	0.011	0.042

CHMP4C, GZMB and TNF.

### Independence Analysis of Prognostic Value of the Panel by Prognostic DEPRGs

The age, grade and International Federation of Gynecology and Obstetrics (FIGO) stage data of CESC patients were extracted from the clinical data in TCGA cohort. Combined with the risk score in the prognostic panel, the independent prognosis was analyzed by univariate and multivariable Cox regression models.

### Gene Ontology, and Kyoto Encyclopedia of Genes and Genomes Functional Enrichment Analyses Between High- and Low-Risk Groups

The CESC patients in the TCGA cohort were divided into the high-and low-risk groups according to the median risk score, and the DEGs in the two groups were screened by using the “limma” R package according to the condition of |logFC| > 1 and FDR < 0.05. The KEGG and GO enrichment analyses of the DEGs were performed with the “clusterProfiler” R package, and the results were shown in a bar chart using the “ggplot2” R package.

### Comparison of ssGSEA Enrichment Scores for Immune Cells and Immune Pathways

After the patients were divided into the high-and low-risk groups according to the median risk score, the differences of immune-related cells and immune pathway activation between the two groups were calculated by single-sample gene set enrichment analysis (ssGSEA) algorithm, and the results were shown with a boxplot by “ggpubr” R package.

### Analysis of Immune-Related Cells in the TCGA Cohort

“CIBERSORT” algorithm was used to score the immune-related cells of CESC patients in TCGA cohort, and the relative content of each immune-related cell in each sample was obtained. The “corrplot” R package was used to show the results of immune-related cell infiltration and the correlation between immune-related cells. The difference of immune-related cell infiltration between normal and CESC patients was analyzed and displayed by “pheatmap” and “vioplot” R packages. The infiltration of immune-related cells of CESC patients was combined with survival data. Kaplan-Meier survival analysis was performed with “survival” and “survminer” R packages after the patients were divided into high-and low-infiltration groups according to the previously calculated median value of each immune-related cell infiltration.

### Correlation of Prognostic DEPRGs and Immune Related Cells

The results of CIBERSORT immune-related cell infiltration were merged with the expression of prognostic DEPRGs, and then the correlation between each prognostic DEPRG and immune-related cells was analyzed, and the results were shown by a lollipop diagram.

### Cell Culture

Normal cervical cell line (Ect1/E6E7) and cervical cancer cell lines (HeLa, SiHa, C33A) were acquired from the American Type Culture Collection (ATCC, Manassas, VA, USA). Ect1/E6E7 was cultured in Roswell Park Memorial Institute (RPMI) 1640 medium (HyClone, USA). DMEM (Gibco, USA) was used to cultivate HeLa and C33A cells, and EMEM (Gibco, USA) was used to cultivate SiHa cells. Each medium was supplemented with 10% fetal bovine serum (Gibco, USA) and all cell lines were cultured in a humidified incubator in an atmosphere of 5% CO_2_ at 37°C.

### RNA Extraction, Reverse Transcription, and qRT-PCR

Total RNA was extracted from cell lines using the RNAiso Plus reagent (TaKaRa, Shanghai, China) and reverse transcribed into cDNA using the reverse transcription kit (TaKaRa) according to the manufacturer’s instructions. mRNA-specific cDNA primers were used in quantitative real-time PCR (qRT-PCR). The QuantStudioTM Design &Analysis Software1.3.1 PCR System was used for all real-time PCR experiments. For quantification, the 2^-ΔΔCt^ method was utilized, and the fold change for the targeted genes was standardized using internal control. The following were the PCR reaction conditions: 95°C for 30 seconds, followed by 95°C for 5 seconds and 60°C for 30 seconds 40 cycles, then 95°C for 15 seconds, 60°C for 1 minute, and 95°C for 15 seconds again. The levels of expression were compared to those of the internal reference gene GAPDH. The primer sequences were listed as below: CHMP4C forward 5′-GCAAGAGATCACAGAGCAACAGGAT-3′, reverse:5′-TGGGAGAGAAGAGGAAGGCACATT-3′; TNF forward 5′- GAGGCCAAGCCCTGGTATG-3′, reverse:5′- CGGGCCGATTGATCTCAGC -3′; GZMB forward 5′- CTTCCTGATACGAGACGACTTC -3′, reverse:5′-CACTGTCATCTTCACCTCTTGT-3′; GAPDH forward 5′- CTGACTTCAACAGCGACACC-3′; reverse: 5′- TGCTGTAGCCAAATTCGTTGT-3′.

### Expression Validation of the Prognostic DEPRGs by Gene Expression Profiling Interactive Analysis Database (GEPIA)

GEPIA (http://gepia.cancer-pku.cn/) is an online database management system for the standardized analysis of massive amounts of RNA sequencing data from the TCGA and GTEx datasets. In GEPIA, the expression levels of three prognostic DEPRGs were validated [num(T) = 306; num(N) = 13].

### Statistical Analysis

R software (version 4.1.1) was used for statistical analysis. A Wilcoxon signed-rank test (false discovery rate, FDR<0.05) was used to screen DEGs, and Benjamini and Hochberg (BH) corrections were applied to adjust p values. The Kaplan–Meier survival analysis was used to estimate the survival of CESC patients based on the DEPRGs panel. Univariate and multivariate survival analyses with the Cox regression model were used to identify independent prognostic markers. The Mann–Whitney test was performed to compare immune cell infiltration and immune pathway activation between the two groups. The box plots were created using the GraphPad Prism 9 program, and the data is reported as mean ± standard deviation, and an unpaired t test was performed to detect the differences in prognostic DEPRGs expression between normal cervical cell line and cancer cell lines. A P-value of less than 0.05 was considered to be statistically significant (p < 0.0001=****, p < 0.001 = ***, p < 0.01 = **, and p < 0.05 = *).

## Results

### Identification of Prognostic DEPRGs and Construction of Prognostic Panel

**Figure 2:**
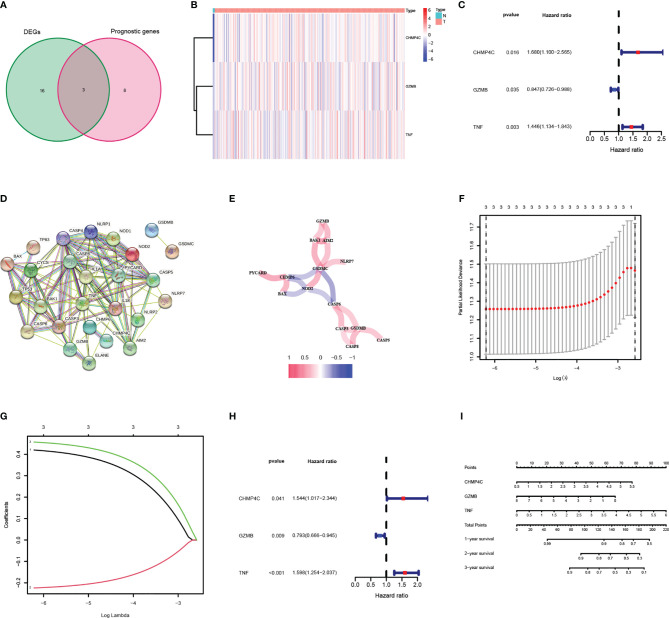
Identification and analysis of DEPRGs in cervical cancer. **(A)** Venn diagram illustrating prognostic DEPRGs. **(B)** Heatmap analysis the expression conditions of prognostic DEPRGs. **(C)** Univariate cox regression analysis for each prognostic DEPRG. **(D)** PPI network showing the interactions of the DEPRGs (interaction=0.4). **(E)** Network analysis of internal correlations among DEPRGs. Correlation coefficients are indicated by different colors. **(F)** LASSO regression analysis of prognostic DEPRGs. **(G)** Cross-validation in the LASSO regression. **(H)** Multivariate cox regression analysis for prognostic DEPRG. **(I)** Nomogram of the prognostic DEPRGs for prediction of the 1-, 2-, and 3-year survival rate.

52 PRGs were extracted from mRNA expression data from TCGA CESC cohort through literature mining, of which 19 genes were differentially expressed in normal and tumor samples, 11 genes were related to the prognosis of CESC patients **(** **Table 2** **)**, and three genes (CHMP4C, GZMB, TNF) intersected together were prognostic DEPRGs **(** **Figure 2A** **)**. The heatmap showed the expression of these three genes at RNA level in each CESC sample and normal sample, and CHMP4C, GZMB, TNF were all upregulated **(** **Figure 2B** **)**. The forest map of univariate COX regression analysis showed that CHMP4C and TNF were high-risk factors, while GZMB was a low-risk factor **(** **Figure 2C** **)**. Then, PPI analysis further explores the interactions between DEPRGs **(** **Figure 2D** **)**. The correlation network of these genes also reveals the internal relationship, with lines indicating co-expression, blue indicating negative correlation and red indicating positive correlation **(** **Figure 2E** **)**. LASSO regression analysis **(** **Figures 2F, G** **)** and multivariate COX regression analysis **(** **Figure 2H** **)** showed that these three prognostic DEPRGs were the optimal genes to construct the prognostic panel. Then a prognostic panel based on these three prognostic DEPRGs was constructed. Based on this panel, the formula for calculating the value at risk of each patient is: Risk Score = CHMP4C × 0.420 + GZMB × (-0.224) + TNF × 0.457. On the basis of this panel, the nomogram for predicting the 1-, 2-, and 3-year survival rate of patients is constructed **(** **Figure 2I** **)**.

### Synthetic Analysis of the Prognostic Value of the Panel by Prognostic DEPRGs

**Figure 3:**
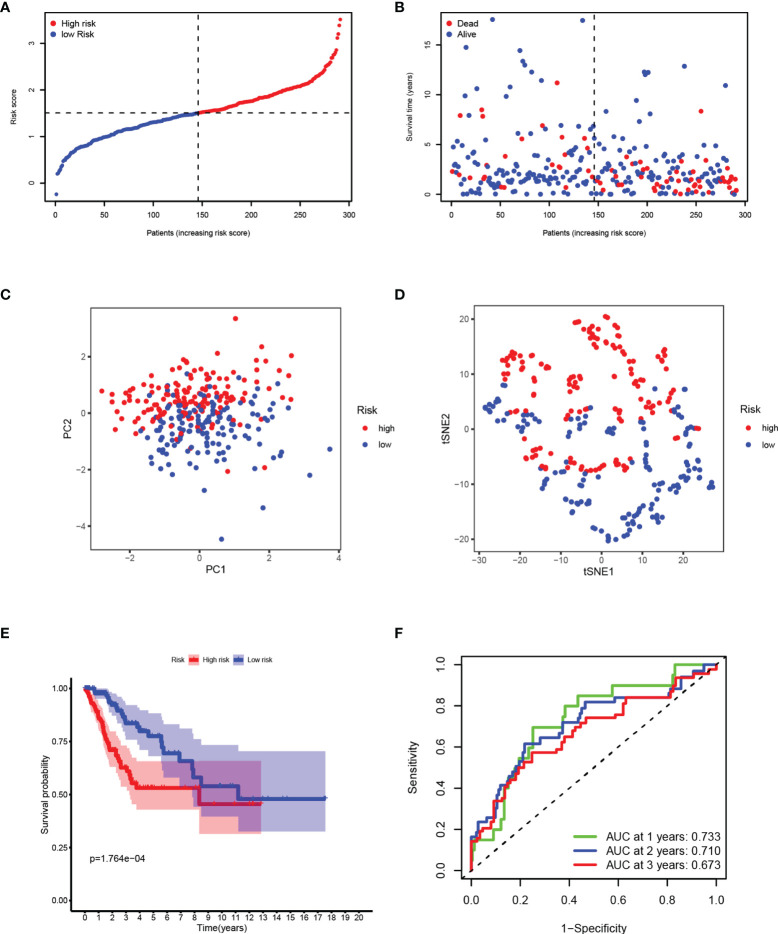
Synthetic analysis of the prognostic value of the panel by prognostic DEPRGs. **(A)** Distribution of samples based on the risk score. **(B)** Score plot for the principal component analysis (PCA). **(C)** Distribution of the risk scores and patient survival status. **(D)** The t-SNE studying the different gene expression patterns of samples. **(E)** Kaplan–Meier curves for patients in the low-and high-risk groups. **(F)** The 1-, 2-, and 3-year ROC curves for survival prediction with AUC values.

After removing the patients with a survival time of 0, based on the median risk score calculated by the established panel, the patients are divided into high-and low-risk groups **(** **Figure 3A** **)**. The patients’ survival rate decreases with the increase of the risk score, reflecting the predictive effectiveness of the panel **(** **Figure 3B** **)**. Through dimensionality reduction processing, PCA **(** **Figure 3C** **)** and t-SNE **(** **Figure 3D** **)** analyses, show that the risk panel can distinguish high-and low-risk patients into two clusters. KM survival analysis shows that the survival rates of patients between the two groups are significantly different, and the survival rate in the high-risk group is significantly lower than that in the low-risk group **(** **Figure 3E** **)**. The area under the ROC curve (AUC) of 1-, 2-, and 3-year are 0.733, 0.710, and 0.673 respectively, which are all around 0.7, indicating that the sensitivity and specificity of the prognostic panel are relatively high **(** **Figure 3F** **)**.

### Independence Analysis of Prognostic Value of the Risk Panel

**Figure 4:**
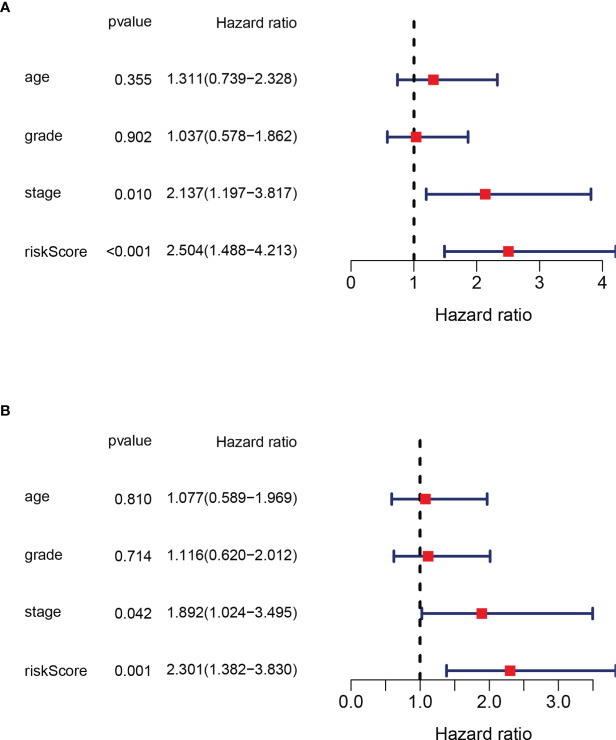
Independence analysis of prognostic value of the panel by prognostic DEPRG. **(A)** Univariate and **(B)** Multivariate COX regression models of the risk score and other clinical characteristics.

In order to verify that the risk score calculated on the basis of this panel can be used as an independent factor predicting the prognosis of the patient, we combined clinical data to analyze in both univariate and multivariate Cox regression models. In univariate COX regression model analysis, the risk score (HR = 2.137, 95% CI: 1.197–3.817, p<0.001) can be used as an independent prognostic factor **(** **Figure 4A** **)**. After adjustment for potential confounders, the multivariate COX regression model analysis shows the risk score (HR=2.301, 95%CI: 1.382-3.830, p=0.001) can still be used as an independent factor predicting prognosis **(** **Figure 4B** **)**. In summary, the risk score is an independent indicator predicting the prognosis of the patient.

### Functional Analysis of DEGs on the Basis of the Risk Panel

**Figure 5:**
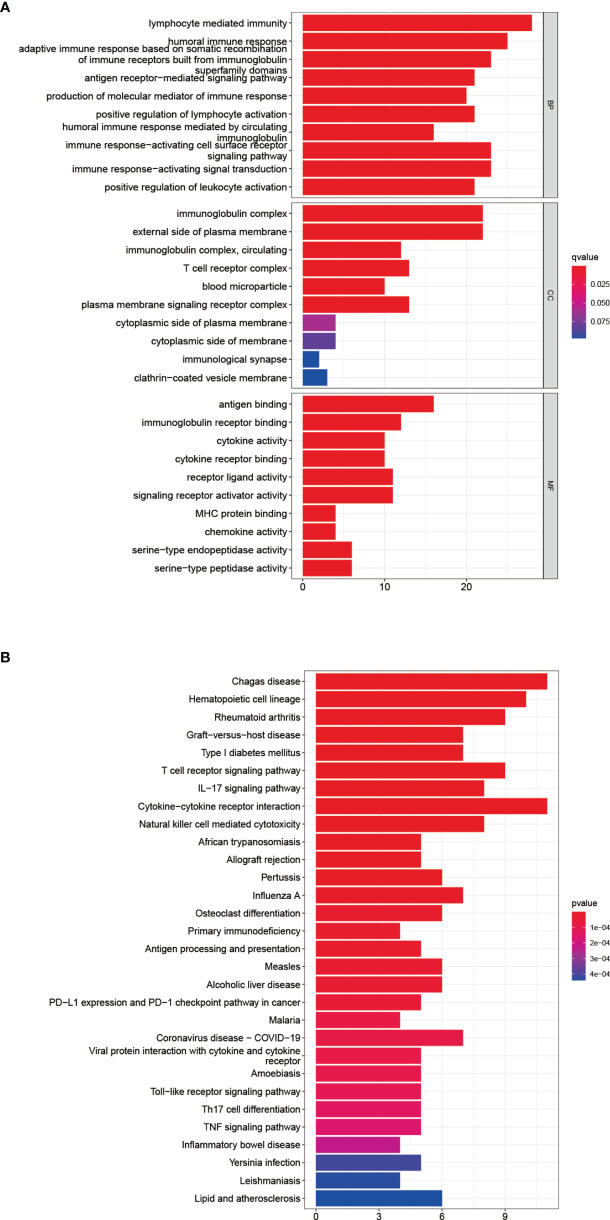
Functional analysis of DEGs. Results of GO **(A)** and KEGG **(B)** pathways enrichment analyses on the DEGs between the two-risk groups in the TCGA cohort.

After dividing patients into high-and low-risk groups based on the risk panel, in order to further explore the differences in gene functions and pathways between the two groups, DEGs were obtained on the condition of |logFC|> 1 and FDR <0.05. Subsequently, these DEGs were analyzed for GO and KEGG enrichment analyses. GO enrichment analysis showed that the biological process (BP) of DEGs was enriched in lymphocyte mediated immunity, humoral immune response, adaptive immune response based on somatic recombination of immune receptors built from immunoglobulin superfamily domains, etc. Cellular component (CC) was enriched in immunoglobulin complex, External side of plasma membrane, immunoglobulin complex and circulating, T cell receptor complex, blood microparticle, plasma membrane signaling receptor complex. Molecular function (MF) was enriched in antigen binding, immunoglobulin receptor binding, cytokine activity, etc. (adjusted p <0.05, **Table 4**, **Figure 5A**). KEGG enrichment analysis showed that DEGs were enriched in Chagas disease, Hematopoietic cell lineage, Rheumatoid arthritis and other pathways (adjusted p <0.05, **Table 5**, **Figure 5B**). The results of GO and KEGG enrichment analyses were also displayed by bubble plots **(** **Supplementary Figure 1** **)**.

**Table 4:** GO analysis.

ONTOLOGY	ID	Description	q value	Count
BP	GO:0002449	lymphocyte mediated immunity	4.02E-24	28
BP	GO:0006959	humoral immune response	1.18E-19	25
BP	GO:0002460	adaptive immune response based on somatic recombination of immune receptors built from immunoglobulin superfamily domains	1.48E-17	23
BP	GO:0050851	antigen receptor-mediated signaling pathway	2.83E-16	21
BP	GO:0002440	production of molecular mediator of immune response	4.05E-16	20
BP	GO:0051251	positive regulation of lymphocyte activation	1.39E-15	21
BP	GO:0002455	humoral immune response mediated by circulating immunoglobulin	2.03E-15	16
BP	GO:0002429	immune response-activating cell surface receptor signaling pathway	2.03E-15	23
BP	GO:0002757	immune response-activating signal transduction	2.03E-15	23
BP	GO:0002696	positive regulation of leukocyte activation	9.33E-15	21
CC	GO:0019814	immunoglobulin complex	5.40E-25	22
CC	GO:0009897	external side of plasma membrane	7.48E-17	22
CC	GO:0042571	immunoglobulin complex, circulating	1.53E-14	12
CC	GO:0042101	T cell receptor complex	2.00E-12	13
CC	GO:0072562	blood microparticle	1.14E-08	10
CC	GO:0098802	plasma membrane signaling receptor complex	1.45E-08	13
MF	GO:0003823	antigen binding	4.73E-16	16
MF	GO:0034987	immunoglobulin receptor binding	1.90E-14	12
MF	GO:0005125	cytokine activity	1.44E-06	10
MF	GO:0005126	cytokine receptor binding	3.98E-06	10
MF	GO:0048018	receptor ligand activity	8.81E-05	11
MF	GO:0030546	signaling receptor activator activity	8.81E-05	11
MF	GO:0042287	MHC protein binding	0.000358	4
MF	GO:0008009	chemokine activity	0.000706	4
MF	GO:0004252	serine-type endopeptidase activity	0.000833	6
MF	GO:0008236	serine-type peptidase activity	0.001269	6

The top 10 results of GO functional enrichment analysis.

**Table 5:** KEGG analysis.

ID	Description	q value	Count
hsa05142	Chagas disease	4.01E-11	11
hsa04640	Hematopoietic cell lineage	5.06E-10	10
hsa05323	Rheumatoid arthritis	5.87E-09	9
hsa05332	Graft-versus-host disease	8.09E-09	7
hsa04940	Type I diabetes mellitus	8.09E-09	7
hsa04660	T cell receptor signaling pathway	8.09E-09	9
hsa04657	IL-17 signaling pathway	7.76E-08	8
hsa04060	Cytokine-cytokine receptor interaction	4.71E-07	11
hsa04650	Natural killer cell mediated cytotoxicity	8.30E-07	8
hsa05143	African trypanosomiasis	4.12E-06	5
hsa05330	Allograft rejection	4.29E-06	5
hsa05133	Pertussis	6.16E-06	6
hsa05164	Influenza A	5.62E-05	7
hsa04380	Osteoclast differentiation	0.000108	6
hsa05340	Primary immunodeficiency	0.000108	4
hsa04612	Antigen processing and presentation	0.000108	5
hsa05162	Measles	0.000144	6
hsa04936	Alcoholic liver disease	0.000154	6
hsa05235	PD-L1 expression and PD-1 checkpoint pathway in cancer	0.000173	5
hsa05144	Malaria	0.000247	4
hsa05171	Coronavirus disease - COVID-19	0.000247	7
hsa04061	Viral protein interaction with cytokine and cytokine receptor	0.000261	5
hsa05146	Amoebiasis	0.000274	5
hsa04620	Toll-like receptor signaling pathway	0.000288	5
hsa04659	Th17 cell differentiation	0.000331	5
hsa04668	TNF signaling pathway	0.000377	5
hsa05321	Inflammatory bowel disease	0.000512	4
hsa05135	Yersinia infection	0.000896	5
hsa05140	Leishmaniasis	0.000897	4
hsa05417	Lipid and atherosclerosis	0.000897	6

The top 30 results of KEGG functional enrichment analysis.

### ssGSEA Analysis of Immune Cells and Immune Pathways

**Figure 6:**
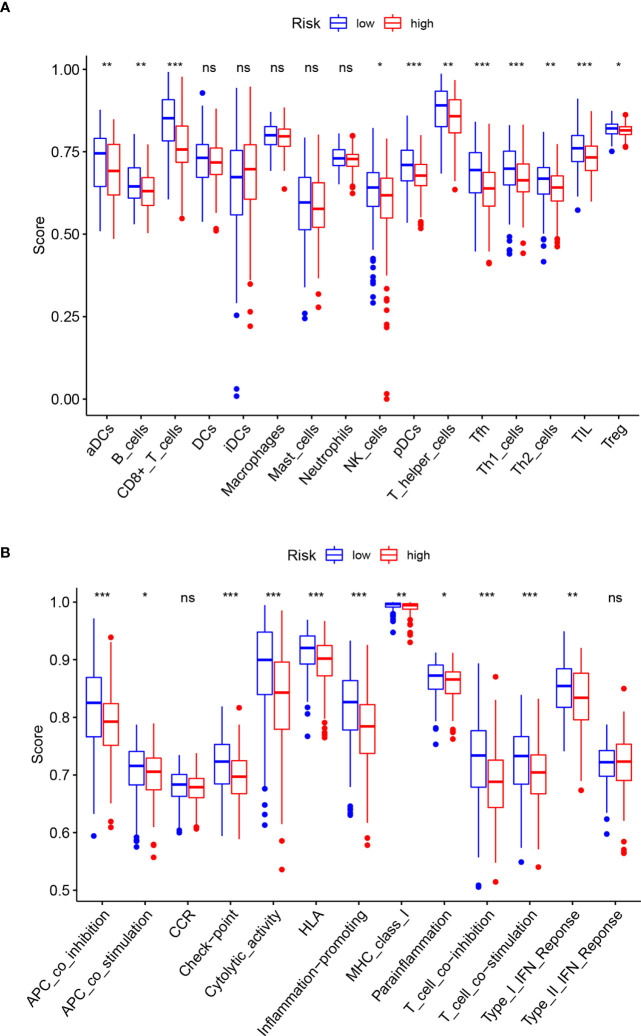
Comparison of ssGSEA enrichment scores for immune cells and immune pathways. **(A)** Comparison of ssGSEA enrichment scores of 16 immune cells between high- (red box) and low-risk (blue box) groups in the TCGA cohort. **(B)** Comparison of ssGSEA enrichment scores of 13 immune-related biological processes between high- (red box) and low-risk (blue box) groups in the TCGA cohort. Adjusted p-values are shown as follows: ns, not significant; *p < 0.05; **p < 0.01; ***p < 0.001.

The ssGSEA analysis performed between the high-and low-risk groups revealed differences in immune-related cells and immune pathway activation. Except for DCs, iDCs, Macrophages, Mast cells, and Neutrophils, which are not statistically significant (P>0.05), the infiltration levels of other immune-related cells in the low-risk group are higher than those in the high-risk group. The most obvious ones are CD8+ T cells, pDCs, Tfh, Th1 cells, TIL (p<0.001) **(** **Figure 6A** **)**. The immune pathways of Type-II IFN Response and CCR are not statistically different between the high-and low-risk groups (P>0.05). Compared with the low-risk group, the remaining immune functions are all low activity in the high-risk group, especially of APC co-inhibition, check point, cytolytic activity, HLA, inflammation promoting, T cell co-inhibition, T cell co-stimulation (p<0.001) **(** **Figure 6B** **)**.

### Analysis of Immune-Related Cells Between CESC and Normal Samples in TCGA Cohort

**Figure 7:**
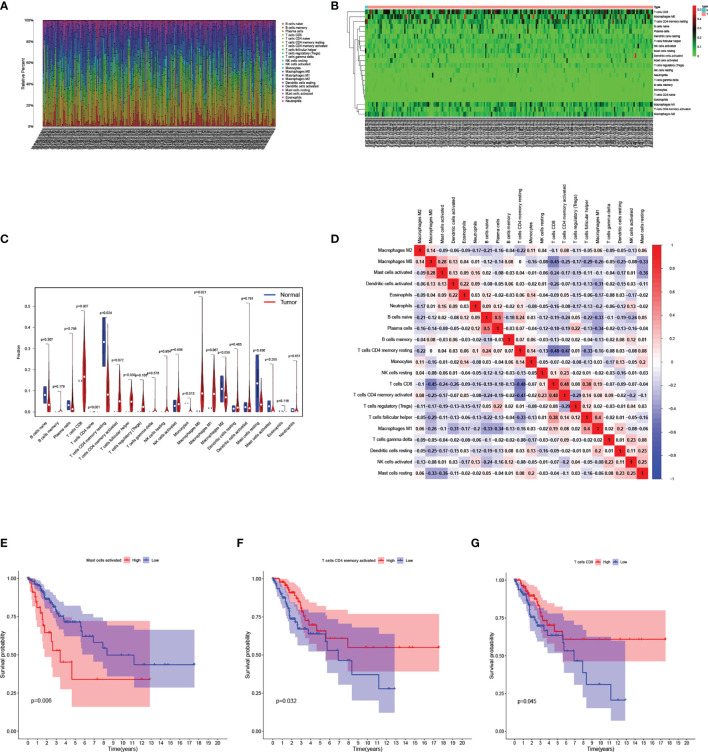
Analysis of immune-related cells in the TCGA cohort. **(A)** Bar chart analysis the quantity of immune-related cells in each sample. **(B)** Heatmap analysis of the quantity of immune-related cells in normal and tumor samples. **(C)** Violin diagram for difference analysis of immune-related cells between normal samples and tumor samples. **(D)** Display of the relationship between immune-related cells. **(E–G)** Kaplan–Meier curves for patients in the low- and high-risk groups of activated Mast-cell **(E)**, activated memory CD4+T cells **(F)**, CD8+T cells **(G)**.

The relative infiltration level of 22 kinds of immune-related cells in each sample was calculated by “CIBERSORT” algorithm. **(** **Figure 7A** **)**. Then the normal samples and CESC samples were separated, and the infiltration level of immune-related cells was represented by a heatmap **(** **Figure 7B** **)**. It was further revealed that in the case of statistical differences (p<0.05), the infiltration levels of naïve CD4+T cells, Monocytes in normal samples was higher than those in CESC samples, while the infiltration levels of resting memory CD4+T cells, follicular helper T cells and M0 Macrophages in CESC samples were higher than those in normal samples **(** **Figure 7C** **)**. In the study of correlation between immune-related cells, it was found that the positive correlation between Plasma cells and naïve B cells was the strongest (correlation coefficient =0.5), while the negative correlation between resting memory CD4+T cells and CD8+T cells, activated memory CD4+T cells was very strong (correlation coefficient=-0.48, -0.47 respectively) **(** **Figure 7D** **)**. Finally, in the survival analysis of the group with high-or low-risk of immune-related cells infiltration, the survival rate of activated Mast cells low infiltration group was higher than that of high infiltration group **(** **Figure 7E** **)**. On the contrary, the survival rate of activated memory CD4+T cells and CD8+T cells infiltration group was higher than that of low infiltration group **(** **Figures 7F, G** **)**.

### Correlation of Prognostic DEPRGs and Immune Related Cells

**Figure 8:**
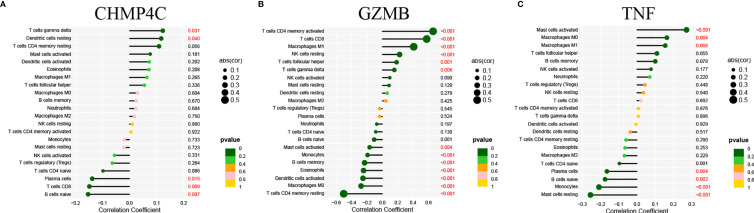
Correlation of prognostic DEPRGs and immune related cells. Lollipop diagram for the correlation between immune related cells and CHMP4C **(A)**, GZMB **(B)**, TNF **(C)**.

We then explore the correlation between the three prognostic DEPRGs and immune-related cells. It is expected to find the relationship between genes and immune-related cells, and then predict the immune status of patients through the expression of prognostic DEPRGs. CHMP4C was positively correlated with gamma delta (γδ) T cells, resting Dendritic cells and negatively correlated with Plasma cells, CD8+T cells and naïve B cells **(** **Figure 8A** **)**. GZMB was positively correlated with activated memory CD4+T cells, CD8+T cells, M1 Macrophages, resting NK cells, follicular helper T cells, gamma delta (γδ) T cells, and negatively correlated with activated Mast cells, Monocytes, memory B cells, Eosinophils, activated Dendritic cells, M0 Macrophages, resting memory CD4+T cells **(** **Figure 8B** **)**. TNF was positively correlated with activated Mast cells, M0 Macrophages, M1 Macrophages and negatively correlated with Plasma cells, naïve B cells, Monocytes and resting Mast cells **(** **Figure 8C** **)**. The specific correlation between prognostic DEPRGs and immune related cells is also represented in detail **(** **Supplementary Figure 2** **)**.

### External Validation of the Expression Levels of the Three Prognostic DEPRGs

**Figure 9:**
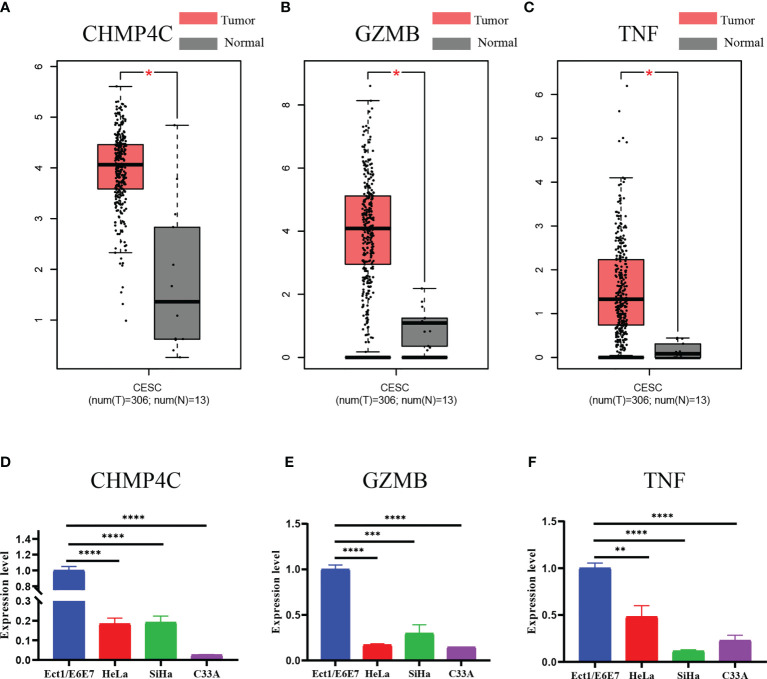
Expression validation of three prognostic DEPRGs. The results of the GEPIA database **(A–C)** for the expression difference between CESC and normal cervical tissues. The results of the qRT-PCR **(D–F)** experiment for the expression difference between normal cervical cell line and cervical cancer cell lines. *p < 0.05; **p < 0.01; ***p < 0.001; ****p < 0.0001.

Finally, we examined the expression levels of the three prognostic DEPRGs using the GEPIA database and a qRT-PCR experiment. The GEPIA database is a merger of the TCGA database and the GTEx database. All 306 tumor tissue samples for cervical cancer were from the TCGA database, while 10 of the 13 normal cervical tissue samples were provided by the GTEx database and 3 by the TCGA database. According to the GEPIA database, CHMP4C, GZMB, and TNF are all strongly expressed in CESC relative to normal cervical tissue **(** **Figures 9A–C** **)**. However, qRT-PCR results revealed that the three prognostic DEPRGs were all weakly expressed in all cervical cancer cell lines as compared to normal cervical cell line, with significant statistical differences **(** **Figures 9D–F** **)**.

## Discussion

Pyroptosis, a newly discovered mode of programmed cell death, and inflammatory bodies, a key substance in its pathway, have been found in a variety of tumor cells (28). In the study of gynecologic malignancies, the prognostic panel constructed by PRGs has been reported in endometrial cancer and ovarian cancer. However, the reports about prognostic panel of PRGs in CC are limited.

In this study, we used mRNA expression data and clinical data of CESC patients from TCGA database. Univariate COX regression, LASSO regression, and multivariate COX regression analyses were used to obtain three prognostic DEPRGs, by which to construct the prognostic panel. Then, independence analysis showed that risk score of this panel could be used as an independent factor to predict the prognosis of CC patients. The predictive markers associated with pyroptosis proposed in our study include three prognostic DEPRGs (CHMP4C, GZMB, TNF). CHMP4C, charged multivesicular body protein 4C, is a member of the chromatin-modified protein/charged multivesicular body protein (CHMP) family. These proteins are elements of the endosomal sorting complex required for transport III (ESCRT-III), which is involved in the degradation of surface receptor proteins as well as the formation of endocytic multivesicular bodies (MVBs) (29). One research found that the enhanced intracellular p53 levels on E6/E7 inhibition were linked to increased expression of both TSAP6 and CHMP4C in HeLa cells, promoting the development of CC (30). Another study found that CHMP4C expression was higher in CC tissues and high CHMP4C expression was associated with lower survival rates. Furthermore, overexpression of CHMP4C induced activation of the epithelial-mesenchymal transition pathway, whereas deletion of CHMP4C inhibited activation. CHMP4C promoted CC cell survival and motility by regulating epithelial-mesenchymal transition (31). In our study, CHMP4C was highly expressed in CC samples and was a high-risk gene, consistent with previous studies (31). GZMB, granzyme B, encodes a protein that is critical in cell-mediated immune responses for the rapid induction of apoptosis in cytolytic T lymphocytes (CTL). It is acknowledged that high level of estrogen is an important risk factor for cervical carcinogenesis. On the one hand, early synergism between HPV16-E7 oncoprotein and 17β-estradiol inhibits granzyme B expression in CC models (32). On the other hand, estrogen strongly induces the expression of human granzyme B inhibitor and protease inhibitor 9 (PI-9). CTL and natural killer (NK) cells induce apoptosis in target cells using the granzyme pathway, and induction of human granzyme B inhibitor and PI-9 expression inhibits CTL and NK cell-mediated apoptosis, which in turn promotes proliferation of CC cells (33). Interestingly, GZMB appears to be a pro-oncogene in our study, as it was upregulated threefold in tumor tissue. A study has shown that high levels of granzyme B expression in invasive cervical carcinoma correlates with a poor response to treatment (34). However, it also contributed to prolonged patient survival as it was enriched in the low-risk group. Given the limited data available from TCGA CESC cohorts and the often contradictory results from different tumors, our results on GZMB provide some insights for further studies. TNF, tumor necrosis factor, encodes a multifunctional pro-inflammatory cytokine, which belongs to the TNF superfamily. TNF-α has not a single effect on tumor cells, but can promote tumor cell proliferation and differentiation, as well as inhibit proliferation and induce apoptosis. For one thing, TNF-α can exert anti-tumor effects by inducing apoptosis, participating in the body’s immune response, and inducing programmed cell necrosis, and has been used clinically as an anti-tumor agent (35). For another thing, it was demonstrated that TNF-α was massively produced by tumor cells. This subsequently activates NF-κB, which originates from the autocrine-paracrine loop produced by TNF-α in tumor cells (36). In addition, TNF-α increases the production of pro-inflammatory cytokines, such as IL-1, IL-6, IL-8 and RANTES, which may produce complications in tumor pathology (37). TNF-α is also involved in tumor cell proliferation by increasing fibrinogen activator inhibitor type 2 (PAI-2), which is assumed to protect cells from apoptosis. Furthermore, it increases angiogenesis by inducing vascular endothelial growth factor (VEGF), which also accelerates the formation of lymphatic vessels leading to lymphatic metastasis of tumor lesions (37). Moreover, TNF-α, as an activator of MMP, is associated with tumor progression (38, 39). It can induce MMP-9, a collagenase that is strongly implanted in tumor invasion and metastasis (40). We found that TNF expression was upregulated in CC tissues and its high expression predicted low survival, suggesting that it acts as a tumor-promoting gene in our study. However, because of the complexity of the relationship between TNF and tumors, the specific tumor-promoting mechanisms need to be further investigated.

After constructing the prognostic panel, CC patients were divided into high-and low-risk groups based on risk score. DEGs from the high-and low-risk groups were used for GO and KEGG enrichment analyses, and the results indicated that DEGs were mainly enriched in immune responses and inflammatory cell chemotaxis, presumably suggesting that pyroptosis is closely related to the regulation of the tumor immune microenvironment. Therefore, we analyzed the differences of immune-related cell and immune pathway activation between high-and low-risk groups using ssGSEA. Except for the results that were not statistically different, the infiltration of immune-related cells and immune pathway activation were significantly higher in the low-risk group than in the high-risk group, and it was hypothesized that the poor survival outcome in the high-risk group might be caused by reduced levels of antitumor immunity (41). Furthermore, we explore the immune-related cell infiltration of CESC patients in the TCGA database. First, the most infiltrated immune cells are CD8+ T cells and macrophages, which are key cytotoxic lymphocytes against cancer and provide immune surveillance for cancer (42). The macrophages include inactivated M0 macrophages, pro-inflammatory M1 macrophages, and immunosuppressive M2 macrophages (43). Macrophages are plastic and heterogeneous immune cells, and in our study, macrophages M0 and M1 were enriched in CC tissue, while M2 were highly infiltrated in normal cervical tissue, which reflects the complexity of macrophages in tumor microenvironment (TME) (44). We found significant differences in the infiltration of immune-related cells in normal tissues and in CC tissues, suggesting that this different infiltration pattern is intrinsic to individual differences in CC patients and has important clinical implications for the diagnosis and treatment of CC (45). In the study of the correlation between immune-related cells, we found that CD8+ T cells were positively correlated with activated memory CD4+ T cells and negatively correlated with resting memory CD4+ T cells, which is consistent with the finding that CD8+ T cells and activated CD4+ T cells are required for the initiation of cytotoxic immune responses (46). Subsequently, patients were divided into high-and low-infiltration groups according to the level of immune-related cell infiltration, and Kaplan-Meier results showed that activated memory CD4+ T cells and CD8+ T cells had a higher survival rate in the high infiltration group than in the low-risk group. These two immune cells are key mediators of protective immune responses, and high levels may prolong patients’ OS (47). It was shown that activated mast cells infiltrate tumor sites and correlate with tumor growth, angiogenesis and clinical outcome (48), which is consistent with our result that OS was lower in the activated mast cell high infiltration group. Furthermore, we investigated the correlation between prognostic DEPRGs and immune-related cell infiltration. CHMP4C and TNF, as high-risk genes for tumor promotion, positively correlate with promoting tumor progression immune-related cells such as activated mast cells and resting dendritic cells, and negatively correlate with protective immune-related cells such as naïve B cells and CD8+ T cells. In contrast, GZMB, a low-risk gene for tumor suppression, was positively associated with protective immune-related cells such as CD8+ T cells, M1 macrophages, and negatively associated with tumor progression immune-related cells such as activated dendritic cells (49). Overall, the correlation between genes and immune-related cells was consistent with existing studies. However, considering the limitations of retrospective study and the complexity of the TME, the specific relationships between immune-related cells and genes need to be further investigated.

Finally, we used the GEPIA database and qRT-PCR experiments to validate the three prognostic DEPRGs in CC. The results of GEPIA database show differences in expression levels in CC and normal cervical tissues, which is consistent with the results of previous analysis. Interestingly, the results of qRT-PCR experiments using normal cervical cell line and cervical cancer cell lines were completely opposite. There are several reasons for this discrepancy: firstly, the original data used to construct the panel in this paper were downloaded from the TCGA database, which is the data of samples, not cellular data; secondly, tumor cells exist in a complex TME (50). The essence of TME is the cellular and non-cellular components present in and around the tumor. Generally, TME is subdivided into extracellular matrix (ECM), stromal cells and immune cells (51). Previous immune-related analyses in this paper showed that CHMP4C, TNF, and GZMB are all closely related to the tumor immune microenvironment. So the expression of these genes may be an overall reflection of the TME, which suggests that the connection between tumor cells and the rest of various cells in the TME is a complex network that deserves further study and exploration.

Although this study made some important findings regarding pyroptosis and CC, there are still some limitations. First, the small number of normal samples in the TCGA database may lead to some bias in the analysis; second, there is no external validation group, which may lead to poor generalization of the panel; and finally, the study is a retrospective study design, which requires a large amount of experimental data to confirm the findings.

## Conclusion

This is a systematic study of the association between PRGs and CC patient prognosis. After establishing a three-gene prognostic marker based on prognostic DEPRGs, we investigated immune-related cell infiltration and the relationship between immune-related cells and prognostic DEPRGs. Our findings shed light on the role of pyroptosis-related pathways in the development and progression of CC. However, investigations into the molecular mechanisms of pyroptosis-related pathways and immunotherapy are limited to date, and further clinical research is required to refine the linked mechanism.

## Data Availability Statement

The original contributions presented in the study are included in the article/**Supplementary Material**. Further inquiries can be directed to the corresponding authors.

## Author Contributions

HH, MY, and WD designed the research and drafting the manuscript, contributed equally to the whole study. JD, BY, and BH participated in the data analysis and results discussion. QZ, FL, and LH were involved in the drafting and critical revision of the manuscript. All authors read and approved the final manuscript.

## Funding

This work was supported by the National Natural Science Foundation of China (No. 81572546; 81972422; 81771529; 81871167; 82071644).

## Conflict of Interest

The authors declare that the research was conducted in the absence of any commercial or financial relationships that could be construed as a potential conflict of interest.

## Publisher’s Note

All claims expressed in this article are solely those of the authors and do not necessarily represent those of their affiliated organizations, or those of the publisher, the editors and the reviewers. Any product that may be evaluated in this article, or claim that may be made by its manufacturer, is not guaranteed or endorsed by the publisher.
