# Supplementary figures and images for: A Pyroptosis-Related Gene Panel for Predicting the Prognosis and Immune Microenvironment of Cervical Cancer
Source: Front Oncol. 2022 Apr 29;12:873725. doi: 10.3389/fonc.2022.873725 (PMC9099437; doi:10.3389/fonc.2022.873725)

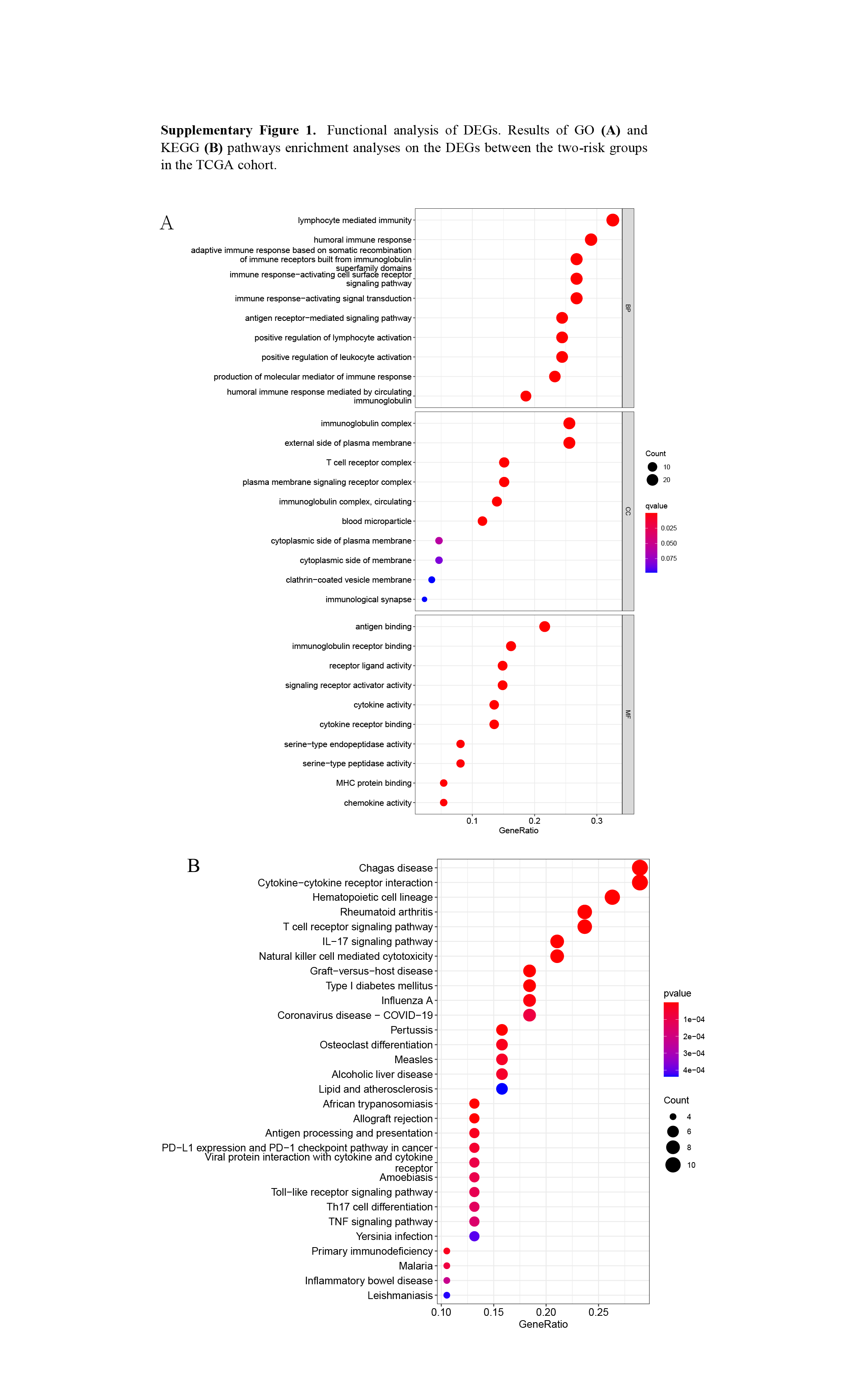

Supplement: Supplementary file 1 [file Image_1.tif]

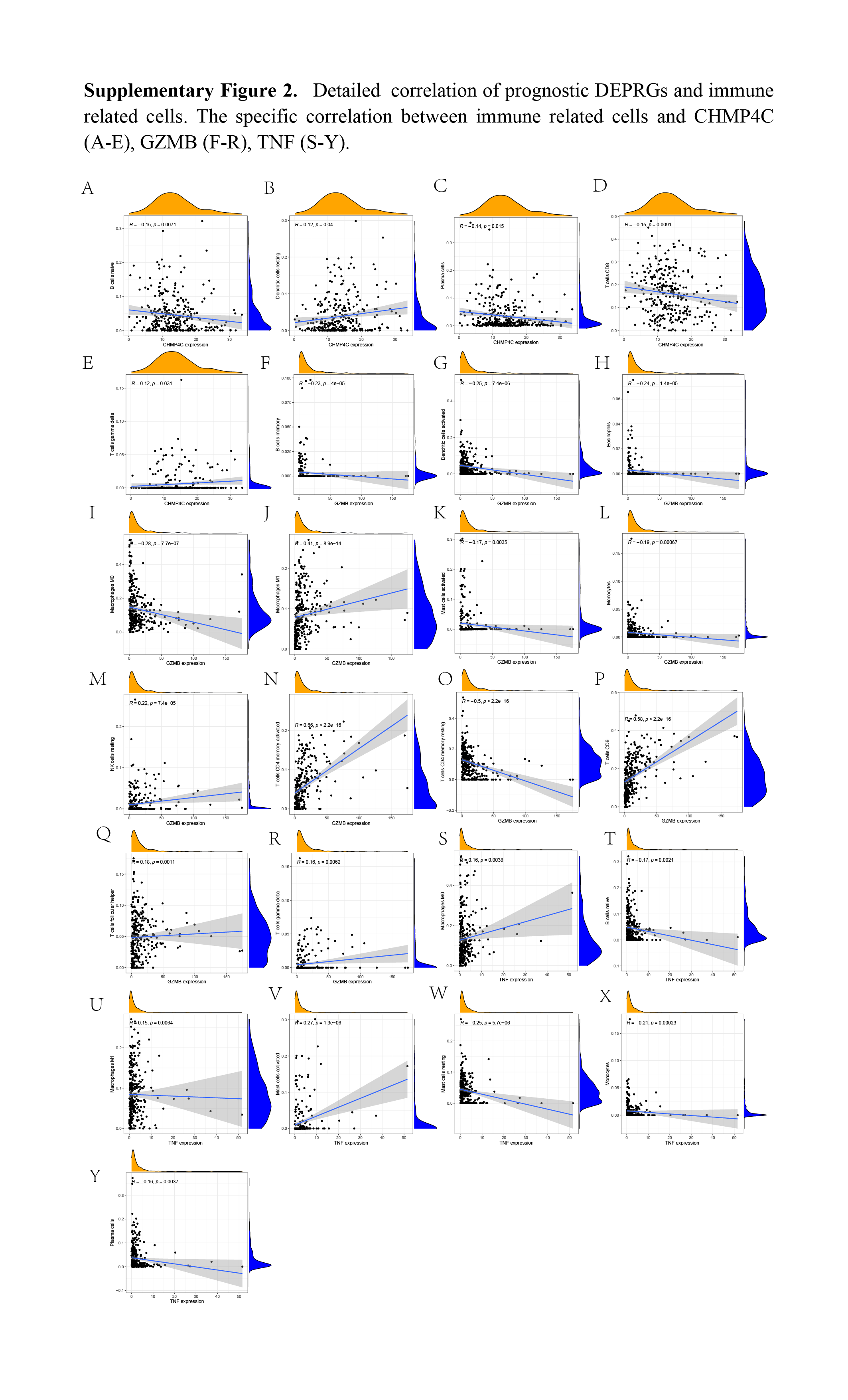

Supplement: Supplementary file 2 [file Image_2.tif]
